# The Design and Development of a Personalized Leisure Time Physical Activity Application Based on Behavior Change Theories, End-User Perceptions, and Principles From Empirical Data Mining

**DOI:** 10.3389/fpubh.2020.528472

**Published:** 2021-02-02

**Authors:** Karlijn Sporrel, Rémi D. D. De Boer, Shihan Wang, Nicky Nibbeling, Monique Simons, Marije Deutekom, Dick Ettema, Paula C. Castro, Victor Zuniga Dourado, Ben Kröse

**Affiliations:** ^1^Department of Human Geography and Spatial Planning, Utrecht University, Utrecht, Netherlands; ^2^Department of Software Engineering, Digital Life Centre, University of Applied Sciences Amsterdam, Amsterdam, Netherlands; ^3^Faculty of Digital Media and Creative Industries, Digital Life Centre, University of Applied Sciences Amsterdam, Amsterdam, Netherlands; ^4^Department of Information and Computing Sciences, Utrecht University, Utrecht, Netherlands; ^5^Faculty of Sports and Nutrition, Amsterdam University of Applied Sciences, Amsterdam, Netherlands; ^6^Consumption and Healthy Lifestyles, Wageningen University & Research, Wageningen, Netherlands; ^7^Department of Health, Sport and Welfare, Inholland University of Applied Sciences, Haarlem, Netherlands; ^8^Department of Human Geography and Spatial Planning, Utrecht University, Utrecht, Netherlands; ^9^Department of Gerontology, Center for Biological and Health Sciences, Federal University of São Carlos, São Paulo, Brazil; ^10^Department of Human Movement Sciences, Federal University of São Paulo (UNIFESP), São Paulo, Brazil; ^11^Faculty of Digital Media and Creative Industries, Digital Life Centre, University of Applied Sciences Amsterdam, Amsterdam, Netherlands

**Keywords:** physical activity, mHealth, Persuasive Technology, behavior change, behavior intervention design, data-mining, reinforcement learning, just-in-time adaptive interventions

## Abstract

**Introduction:** Many adults do not reach the recommended physical activity (PA) guidelines, which can lead to serious health problems. A promising method to increase PA is the use of smartphone PA applications. However, despite the development and evaluation of multiple PA apps, it remains unclear how to develop and design engaging and effective PA apps. Furthermore, little is known on ways to harness the potential of artificial intelligence for developing personalized apps. In this paper, we describe the design and development of the Playful data-driven Active Urban Living (PAUL): a personalized PA application.

**Methods:** The two-phased development process of the PAUL apps rests on principles from the behavior change model; the Integrate, Design, Assess, and Share (IDEAS) framework; and the behavioral intervention technology (BIT) model. During the first phase, we explored whether location-specific information on performing PA in the built environment is an enhancement to a PA app. During the second phase, the other modules of the app were developed. To this end, we first build the theoretical foundation for the PAUL intervention by performing a literature study. Next, a focus group study was performed to translate the theoretical foundations and the needs and wishes in a set of user requirements. Since the participants indicated the need for reminders at a for-them-relevant moment, we developed a self-learning module for the timing of the reminders. To initialize this module, a data-mining study was performed with historical running data to determine good situations for running.

**Results:** The results of these studies informed the design of a personalized mobile health (mHealth) application for running, walking, and performing strength exercises. The app is implemented as a set of modules based on the persuasive strategies “monitoring of behavior,” “feedback,” “goal setting,” “reminders,” “rewards,” and “providing instruction.” An architecture was set up consisting of a smartphone app for the user, a back-end server for storage and adaptivity, and a research portal to provide access to the research team.

**Conclusions:** The interdisciplinary research encompassing psychology, human movement sciences, computer science, and artificial intelligence has led to a theoretically and empirically driven leisure time PA application. In the current phase, the feasibility of the PAUL app is being assessed.

## Introduction

Individuals living in western countries are increasingly inactive (1), which can lead to numerous serious health issues (2). A promising method to increase physical activity (PA) are mobile health (mHealth) interventions, such as smartphone PA applications (3–5). Smartphones are well-integrated into people's lives, enable low-effort continuous activity tracking, and can provide continuous feedback throughout the day (6). Furthermore, with the rapid evolving technological advances in artificial intelligence, enormous potentials arise for the development of more personalized and adaptive apps (7).

The potential of mHealth apps to increase PA among adults has not gone unnoticed, as a large variety of mHealth apps has been developed and evaluated the last decade (3, 4, 8). For instance, several apps have been developed that aim to increase walking by means of a step count app [e.g., (9, 10)] or step count apps with additional text messages [e.g., (11)]. Yet, while some mHealth apps successfully increase PA [e.g., (11, 12)], others do not [e.g., (13, 14)]. It is not well-understood why this is the case (4).

It is difficult to pinpoint the cause of this discrepancy in the effectiveness, since mHealth apps are not always described in sufficient detail (15). Hence, it is unclear what the “active ingredients” (16) of the apps are (such as feedback and goal setting) and how these are operationalized in the app. Furthermore, it is not clear which development methods and processes are effective for designing apps. To illustrate, some development processes place a large emphasis on involving the end user in the process (17), while others draw heavier on the theoretical embedding of the mHealth intervention (18, 19). To gain more insight into how to design and develop effective apps, mHealth researchers are called upon to report their intervention characteristics and the development process in more detail (20, 21).

### Developing mHealth Interventions

Developing mHealth applications is a complex undertaking, involving many stakeholders (including the end users), working within technical and time limitations, and making difficult decisions regarding the practical implementation of theories in the app designs [i.e., the “theory intervention gap” (22)]. To aid the development of effective mHealth interventions, numerous frameworks have been proposed [e.g., (20, 22–25)]. These frameworks often differ in approach. For instance, some frameworks provide a step-wise instruction to develop an mHealth intervention [e.g., the Integrate, Design, Assess, and Share (IDEAS) framework (20) and the MRC framework (23)], while others provide guidance for specific steps such as the translation of behavioral theories to persuasive strategies [e.g., the behavioral intervention technology (BIT) model; (24)].

Although the frameworks describe different developmental processes, there are certain steps that are common in the frameworks. First, several frameworks stress the importance of involving potential end users in different phases of the development process, in order to enable the researchers to integrate these needs in the intervention (20–22, 26). Furthermore, feasibility testing prior to large-scale effect studies is recommended not only to gain insights into the usability and acceptability of the app but also to explore if the users enjoy using the app and its individual persuasive strategies (20–22, 26).

Moreover, most, if not all, frameworks argue that mHealth interventions should be grounded in theory. In other words, the intervention should be based on an understanding of the target behavior and the determinants that constitute behavior (change). Subsequently, the intervention should be designed to target these determinants of behavior. By understanding how apps are designed to change behavior, we can better determine the working mechanisms of the mHealth intervention. Therefore, we will discuss the determinants that constitute PA below.

### Conceptual Model for PA Behavior

There are many factors that underlie PA behavior, such as goals (27–29), motivation (30), emotions (31), habits (32), perceived risk (33), and various factors that lie outside of the individual (34). These determinants of behavior are described in many different theories, but the abundancy and fragmentation of behavioral theories make it difficult to apply them in an mHealth app (35). In a pursuit to design a general model that captures this wide range of factors, the capability, opportunity, motivation, and behavior (COM-B) model was developed (25). The COM-B offers a practical starting point for intervention design that can be enriched by specific theories such as the Self-Determination Theory (30, 36), socio-ecological models (34), goal-setting theory (27–29), and behavior economics (37). The COM-B posits that to engage in a behavior (B), individuals must be physically and psychologically capable (C), their social and physical environment has to offer the opportunity (O), and they have to be motivated (M). The model holds a very broad definition of motivation, which includes all automatic processes of behavior, such as emotions and impulses, as well as conscious processes such as intention and choice.

In addition to the COM-B model, Fogg's Behavior Model (FMB) (38, 39) argues that the individual will not automatically perform the behavior if the opportunity, capability, and motivation are high enough^1^. Rather, according to the FMB, the individual will then be in a “moment of opportunity,” in which the individual can be persuaded to engage in PA if she received a trigger. A trigger can be external, such as a notification or seeing somebody else performing sports. Triggers can also be internal, such as feeling restless or bored. In mHealth interventions, providing an external trigger at “a moment of opportunity” has been coined as just-in-time (adaptive) interventions (JITAIs) (7).

**Figure 1:**
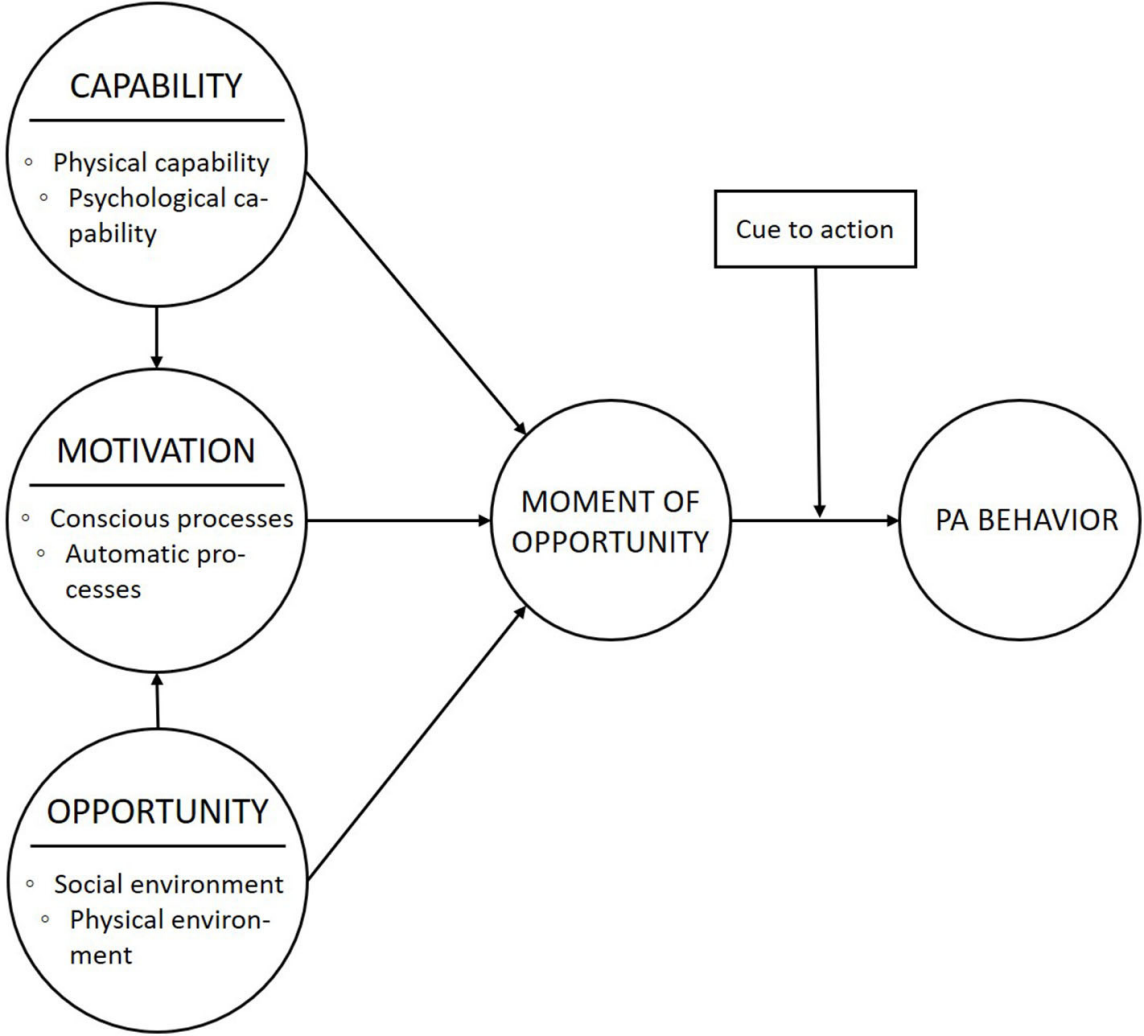
The conceptual model on the main factors and relationships between factors that constitute PA behavior.

Based on the COM-B model and the FBM, we developed a conceptual model that describes the main concepts of interest and pathways leading to the target behavior, namely PA (Figure 1). We expect that when the capability, motivation, and opportunity of individuals to engage in PA is high, a “moment of opportunity” arises. If the participant receives a cue to action at such a moment, she will engage in PA behavior.

### Objective

This current study sets out to develop an mHealth intervention based on a conceptual model to motivate individuals to engage in more PA. In this paper, we describe how we used principles from behavior theory, data-mining techniques, and input from end users and other stakeholders for the development of the Playful data-driven Active Urban Living (PAUL) app. Furthermore, we describe the design of the PAUL app, including a detailed description of the modules of the app and its functionality.

## Method

Our method to develop the PAUL app was based on different development frameworks for mHealth interventions. The behavior change wheel was used to provide a theoretical foundation of the app and to gain a deeper understanding of how the app intents to facilitate behavior change (25). Furthermore, based on the suggestions from the IDEAS framework, we incorporated the needs and wishes of end user in different phases in the design process and we will perform a feasibility study (20, 23). Lastly, the BIT model (24) was used to guide the practical implementation and design of the persuasive strategies in the app.

**Figure 2:**
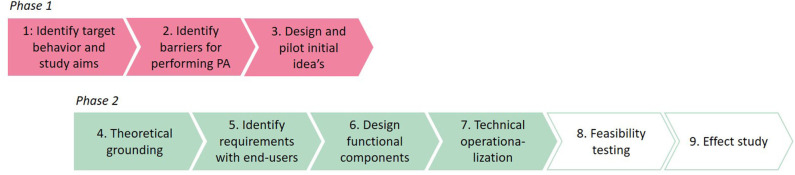
The two-phased development process of the PAUL app.

The development of the PAUL app consists of two phases (Figure 2). It started as a 1-year research project, in which a first version of the app was designed. The aim of this 1-year project was to explore whether location-specific information on performing PA is an enhancement for a PA app. After this 1-year project, we continued the development of the app in a larger project (phase 2), allowing for a more structured development and evaluation of the app. Nine steps were taken to design the PAUL app, of which steps one to three were part of phase one of the project, and steps four to nine are part of the second phase.

Steps 1–5 are described in the *METHODS* section and steps 6 and 7 in the *RESULTS* section. Step 8 is currently being carried out, and the results will be presented in a separate paper. Step 9 is planned after the feasibility study, under the condition that the PAUL app is a feasible intervention to increase PA.

### Specify the Target Behavior and Intervention Aims (Step 1)

The project originated from a request from the municipality of Amsterdam, with a call for novel initiatives that include innovative technologies to motivate individuals to engage more in PA around certain parks of Amsterdam (Oosterpark and Sloterplas). During exploratory discussions with the municipality, it became clear that the municipality was especially interested in (1) using smartphone applications in combination with beacon technology^2^ and (2) interventions that were accessible and affordable for a large group of residents that could benefit from performing more PA (i.e., individuals that are not meeting the PA guidelines). Hence, the intervention should include PA behavior that can be performed by most individuals without having to perform a course or buying expensive materials (i.e., running and walking).

After the discussion with the municipality, we performed explorative interviews to determine the needs and wishes of potential end users. Through snowball recruiting, we included 10 adults who were between 18 and 65 years, who owned a smartphone, and who lived close to the parks (less than a 5-min bike ride). Participants living close to these parks were targeted because of the municipality's requirement to develop an intervention to make this area more attractive for performing PA. Both the ratio of men and women and age distribution were taken into account when recruiting participants. All participants were informed about study participation and signed an informed consent prior to the interviews.

The interviews were performed according to a topic list that included questions on the types of PA they are performing and/or they would prefer to perform, experiences with exercise applications, perceptions of the Oosterpark, the motivations and barriers to engage in PA, and how application functionalities could help them to overcome the barriers and also which barriers they perceived for performing PA and how they could be surpassed (see step 2). The interviews were recorded and transcribed by one researcher. Coding was done manually and both deductive and inductive coding was used.

Five females and five males (between 24 and 59 years) were interviewed. Most engaged in regular active transport (*n* = 8), but they did not engage in leisure time PA (*n* = 8). During the interviews, participants explained that merely running or walking in the park gets boring very quickly. Hence, they proposed to include other activities as well. Bootcamp exercises or strength exercises were mentioned frequently as an addition to running or walking.

Building on the input from these stakeholders, we specified walking, running, and performing strength exercises as the target behavior of the PAUL app. Our project goal is to develop an engaging application with beacon technology that users would like to use on a regular basis (usage aim) to eventually increase their recreational physical activities. By doing so, we aim to improve the general health of individuals (clinical aim). The overarching research aim of the PAUL project is to investigate whether PA applications can increase recreational PA of inactive urban residents. To this end, we aim to explore which designs of applications are more likely to increase PA, how we could implement machine learning to design adaptive and personalized interventions, and to what extent the built environment influences the PA behaviors with the app. Furthermore, we aim to explore to what extent the different cultural contexts of Brazil and the Netherlands influence the views of individuals on exercise apps and the effectiveness of the app.

### Identify Barriers for Performing PA (Step 2)

During the interviews described in step one, we also examined the perceived barriers of inactive individuals that hinder the performance of regular PA. By doing so, we could explore which determinants of behavior (i.e., opportunity, capability, or motivation) are insufficiently present for potential end users, and thus, which determinants of behavior the app should intent to change. To determine which barriers participants' experience, inductive codes were constructed. Then, axial coding was applied in which codes that were related were merged into overarching codes (i.e., barriers). Four key barriers were identified, namely, (1) a lack of time, (2) lack of motivation, (3) a lack of knowledge on performing strength exercises (other than walking or running), and (4) environmental barriers.

#### Time Barrier

The most frequently reported barrier was lack of time. Earlier studies showed that a lack of time could indicate that there are factors that lie outside the participants that interfere with the activity (i.e., lack of opportunity), but it could also be caused by an inability to plan their leisure time or that they simply prefer to do other activities that are more fun or easier to perform [i.e., lack of motivation; (40, 41)]. During the interviews, individuals explained the time barrier was mainly caused by the latter two points. They explained that they had limited time to exercise next to working or studying, in which they preferred to engage in other activities, such as social activities.

“*I like to do it [sport], but it costs a lot of effort and time*.” (P8)

Thus, it appears that the time barrier is related to a lack of motivation. To overcome this barrier, participants indicated that it could help to receive reminders to engage in PA. Yet, they highlighted that they would not appreciate poorly timed reminders. Rather, the reminders should be sent when they have the opportunity to exercise.

#### Motivational Barrier

Participants also mentioned that they simply did not enjoy sporting as much, which did not motivate them to perform PA.

“*I wouldn't say it's the most fun thing to do, the most fun thing to do is just sitting on the couch and watching a TV series or something*.” (P4)

To overcome this barrier, participants proposed to include a functionality that enables them to track their progress and to increase the variety in a PA session by including other exercises, such as doing bootcamp exercises during running or walking. Various participants also mentioned that PA would be more fun if they can do it together with others or when game elements were included, such as a competition. However, although some participants were enthusiastic about social and game elements, others were not.

#### Knowledge Barrier

Even though participants indicated that they would like to perform strength exercises, they explained that they did not know how to perform them. Hence, it seems that there is an insufficient psychological capability of participants to perform these exercises. To overcome this barrier, individuals proposed to include instruction videos on how they can perform these exercises in public spaces.

“*Uhm well I think that if there are outdoor fitness gyms or if you have to do strength exercises, that I would like to see a video first of how I should do that on the device, you know, because sometimes I am a bit scared that you, uhm, afraid or not, but that you misuse the device or perform the exercises wrong, or something, so that you do it all for nothing or that you get an injury*.” (P4)

#### Environmental Barriers

During the interviews, participants also explained that they would not like to sport in the Oosterpark during the evening, because of safety concerns. Some also mentioned other environmental barriers, such as a lack of sports facilities and crowdedness of the park.

“*I did go for a run there once in the morning, but during the day it is often too crowded.”* (P3)

To overcome this barrier, individuals proposed to improve the lighting in the park, to strengthen control and enforcement activities, and to place more sports facilities in the park.

### Design and Pilot the First Version of the App (Step 3)

Based on the input from all stakeholders, a first version of the app was developed. The app included goal setting functionality, feedback during PA, a history view, real-life rewards, and instruction videos to perform the strength exercises. The bootcamp videos were location specific, that is, the participant received an instruction video of somebody performing the strength exercise at the current location of the participant. In the video, the instructor made use of facilities which were already present in the park (such as a bench), to demonstrate how the physical environment can be used for correctly performing strength exercises. Since the videos had to be very location specific, beacon technology was used (see the *Hardware and Software Architecture* section for more details).

After the development of the app, an informal evaluation was performed to explore if an application combined with beacon technology could be used to motivate individuals to perform strength exercises. Residents living around the Oosterpark were invited to test this app for 10 weeks (*n* = 12). Afterwards, participants were interviewed to examine the experience with the app and its functionalities. During the interviews, the participants explained that they enjoyed the instruction videos the most, because it provided a nice alteration with running or walking and it was a novel functionality. However, sometimes, the exercises were too soon after each other, resulting in limiting running and walking time.

Based on this study with the first version of the app, the first design requirement for the PAUL app was drafted:

*Provide JIT instruction*. Individuals should receive instruction videos on performing strength exercises during running or walking, with a 5-min time interval. The instruction videos should provide information on (1) which exercises to perform, (2) how to perform the exercise, (3) where, and (4) when the exercise can be performed. We hypothesize that instruction videos increase feelings of capability, as people can learn through watching others performing the behavior [also known as observational learning; (30)].

### Theoretical and Empirical Grounding of the PAUL App (Step 4)

After establishing that location-specific instructions on performing strength exercises can be a valuable addition to an exercise app, we continued with developing the other modules of the app. To this end, we first examined findings of other PA mHealth studies and theories of behavior change. The reason for this is 2-fold. First, the findings in steps 1–3 were based on small and informal studies, which could lead to biased conclusions regarding effective app modules. Second, as indicated in the *INTRODUCTION*, using a theory gives the researchers tools for moving beyond intuition to design and evaluate mHealth apps based on understanding of behavior.

The conceptual model (Figure 1) explains that several determinants constitute behavior change. To target these determinants, interventions incorporate persuasive strategies or “active ingredients” (16). Persuasive strategies can be defined as “theoretically underpinned elements of an intervention intended to foster a positive behavior or attitude change toward PA,” such as goal setting or providing feedback. In order to select (and design) persuasive strategies that target a particular determinant, behavioral theories and insights from previous studies can provide guidance (25, 42). For instance, it is theorized that the psychological capability of individuals can be increased by “providing information on where and when to perform the behavior.”

Based on three reviews directed at (1) mHealth PA interventions, (2) adults, and (3) the effectiveness of persuasive strategies (3, 4, 8), we identified the most-likely effective persuasive strategies for increasing PA. In a meta-analysis, Eckerstorfer et al. (8) found that mHealth apps including behavioral goals and/or self-monitoring strategies are likely to increase PA, whereas information on where and when or instruction on how to perform the behavior are not. Interestingly, this latter finding is in contrast to what the potential end users stated in steps 2 and 3. The narrative review of Sullivan and Lachman (3) also described that not only strategies supporting self-regulation skills are most likely effective for increasing PA, including self-monitoring and goal-setting strategies, but also feedback, rewards, social support, and coaching strategies (e.g., sending reminders). In contrast, the systematic review of Stucky et al. (4) concluded that there were no persuasive strategies clearly more superior to others for mHealth apps. For instance, their results indicate that PA apps that included “goal setting” were not more effective than interventions without this strategy.

A possible explanation for the findings of Stucky et al. (4) is that the same persuasive strategy is implemented and designed differently across mHealth interventions (43–46). To illustrate, regarding the use of “social media” in mHealth interventions, 36 different design characters have been identified (44). Next, Schembre et al. (43) also found that the technical implementation of “feedback” varies greatly in PA mHealth interventions (i.e., frequency, timing, and mode of delivery). These diverse design characteristics and technical implementations (in the remainder of this paper, we will refer to this as “operationalizations”) might influence the effectiveness of the specific persuasive strategy.

Thus, in the next step, we explored which operationalizations seem most effective (47) regarding the strategies as identified in the previous step:

1) Reminders to engage in PA
2) Goal setting
3) Monitoring of behavior
4) Rewards
5) Social support.

We performed a snowball and gray literature search to identify literature that evaluated the persuasive strategies in experimental trials (e.g., randomized controlled trial and pre-/post-test). Subsequently, the study methods, implementations, and designs of persuasive strategies and study results were systematically extracted. The results of this review (47), the review of Schembre et al. (42), and theories on behavior change resulted in the following operationalization considerations regarding the identified persuasive strategies.

#### Operationalization Considerations for Including Reminders

Reminders are theorized to increase the persuasiveness of the intervention when the content of the message is meaningful to the individual, which in turn can increase one's motivation (25). Moreover, reminders can function as a “cue to action” to initiate behavior in at “the right time” (38, 39). Our scoping review (Sporrel et al., under review) identified only one study that examined the effect of timing of reminder messages in a PA app that demonstrated that optimized timed reminders were perceived as more useful then randomly timed messages, but this does not necessarily result in more PA (48). Additional support for including JITAI reminders can be found in the larger field of preventive healthcare (7). Concerning the content of the reminders, PA apps should aim to include personalized messages (45) as opposed to generic messages [cf. (14, 49)]. To optimize timing or content of the reminders, machine learning methods have been presented (50, 51).

#### Operationalization Considerations for Including Goal Setting

Goal setting can be incorporated in a PA app to increase the motivation of participants (27–29). The results of the scoping review (Sporrel et al., under review) suggest that a PA app should include goals that are challenging [e.g., (52)], personalized to the physical capabilities and/or the context of the individual [e.g., (14, 53)], and set by a system or expert rather than self-set by the individual (54). This latter finding is inconsistent with the goal-setting theory that posits that goals are likely more effective when they are self-set, either on their own or with the help of others. Therefore, a goal-setting function that combines both self-set and assigned goals [i.e., participatory set goals (28)] should be considered.

#### Operationalization Considerations for Including Monitoring and Feedback

The inclusion of feedback in an intervention likely increase both motivation and capability, since providing feedback can elicit positive feelings [motivation; (29)] and increase one's self-efficacy [psychological capability; (30)]. Feedback should be continuously available, personalized, and “actionable” (i.e., information on the amount of PA the user must do to reach her goal) (41). Both messages and visualizations (such as graphs) can be used to provide feedback in an app (Sporrel et al., under review). To provide feedback, the behavior of the individual must be monitored. Previous studies indicate that monitoring PA in mHealth interventions can be done by a single device, since additional devices (such as wearables) are not likely not increase PA behavior (9). However, little is known about the preference for self-monitoring or automatic tracking, nor for the types of behavior (outcomes) that should be tracked (Sporrel et al., under review).

#### Operationalization Considerations for Including Rewards

A reward can be included in an intervention to increase the (extrinsic) motivation of individuals (25, 30, 36, 55). Rewards should be cumulative (e.g., with enough points the participant receives a badge) and given immediately after attaining the goal [cf. (37, 56)].

#### Operationalization Considerations for Including Social Strategies

Social support is argued to be a strong motivator of PA behavior [e.g., (30)]. Yet, the results of the scoping review (Sporrel et al., under review) suggests that multiple operationalizations of social support strategies do not increase the intervention effectiveness [e.g., (9, 12, 57, 58)]. Notably, most of these studies included a small sample size and should therefore be treated with caution. Furthermore, since participants in step 2 and several theories of behavior change suggest including social strategies, we decided to further explore options to include social strategies in the PAUL app.

### Identify Intervention Requirements (Step 5)

After making the selection of the promising persuasive strategies and drafting the operationalization considerations, the considerations were evaluated by potential end users in a focus group study to determine if these considerations are in line with the needs and wishes of our target group (Nibbeling et al., submitted). Five focus groups of 1.5 h (with a 15-min break) were performed with 25 participants in total. The study methods and procedures were approved by the ethics review board of Geosciences of the Utrecht University (reference number: Geo S-19216). The findings of this focus group study informed the requirements that are listed below. The technical implementation of the strategies is provided in step 6.

Participants were recruited though Facebook campaigns and through community centers in Amsterdam. Participants were eligible when they own a smartphone, are native Dutch speakers, if they are in the contemplation or precontemplation phase for engaging in sufficient PA (59), are physically able to perform running and/or walking activities (self-reported), and are aged between 35 and 55. The focus group started with a short PowerPoint presentation on the PAUL project and examples of exercise applications were given. Then, a topic list was used to discuss the six promising persuasive strategies. Participants received a €20 voucher after completing the study. The video recordings of the focus groups were transcribed verbatim and coded and analyzed with ATLAS.ti version 8.0 (ATLAS.ti Scientific Software Development GmbH, 191 Berlin, Germany). The steps described in the framework approach was used to analyze the data (60). Of the 25 participants, most were female (*n* = 17), were in the contemplation phase (*n* = 20), and had experience with exercise applications (*n* = 16).

#### Requirement 1: Reminder

The system should not send too many reminders and, since individuals differ in their optimal timing for reminders, the system should personalize the timing at an individual level. The content of the message should be fun, motivating, friendly, and related to the individual's previous activities, her goal, or the weather.

“*It [the reminder] must therefore be personal and realistic in a friendly way*.” (P1)

#### Requirement 2: Goal Setting

In contrast to the literature, potential end users explained that easy goals are more motivating than difficult goals.

“*Yes, an example of a goal can be: “try to walk for 10 consecutive minutes today” I think that is a little more realistic if you want to motivate people. That people then say: I can do that… Instead of immediately want to take a million steps*.” (P2)

To address this discrepancy between the literature and the participants' wishes, a goal-setting module should have the opportunity to provide both difficult (i.e., run 30 min) and easy goals (i.e., walk 15 min). Additionally, individuals preferred having a large goal (in the future) and sub-goals that help them to attain the future goal. Individuals should be able to set their own goal, but at the same time, they also need to receive suggestions regarding their goal.

#### Requirement 3: Monitoring and Feedback

The app should track and provide feedback on simple PA metrics (time, distance, and calories burned) that are consistent with the metrics of the goal and that can thus relate to the progress toward their goal.

“*And what is also nice is if you, if you set a distance or a goal that the app after expiration, if you stop the app that indicates that: “you have ‘so much %’ of your goal or ‘so many miles’ to go*.” (P2)

Individuals prefer positive, personalized audio feedback during the run (that can be turned off) and a long-term overview of their activities, preferably by a graph or table.

#### Requirement 4: Rewards and Praise

The app should provide a reward for attaining a goal. A complement or positive audio tune was preferred by most individuals, while the views on different virtual rewards such as points and trophies differ greatly. Therefore, a simple “compliment” should be given.

“*Well I think a cub is a bit childish. Just like in the old days, such a teacher sticker in classroom or something. I like it when you do something, more feedback like; you are on track or you are very well on your way to your goal or something*.” (P19)

#### Requirement 5: Social Strategy

In line with the findings of the literature study but in contrast to earlier statements of individuals of the target group (step 2), many potential end users did not think that sharing their results, having a competition, or meeting new people through the application would motivate them to engage in more PA with an app.

“.. *but I would not meet with a ‘Paultje’ [other app user] and start walking with you*.” (P6)

Since both the literature study (step 4) and the results of this focus group suggests (step 5) that including social strategies in PA apps do not necessarily increase or motivate PA behaviors, we decided to not include them in the PAUL app.

#### Identifying Requirements for Healthy Brazilian Adults

To explore if the same requirements apply in a different cultural and environmental setting, the same focus group study has been performed in Brazil. The results of this study are currently being analyzed.

## Results

**Figure 3:**
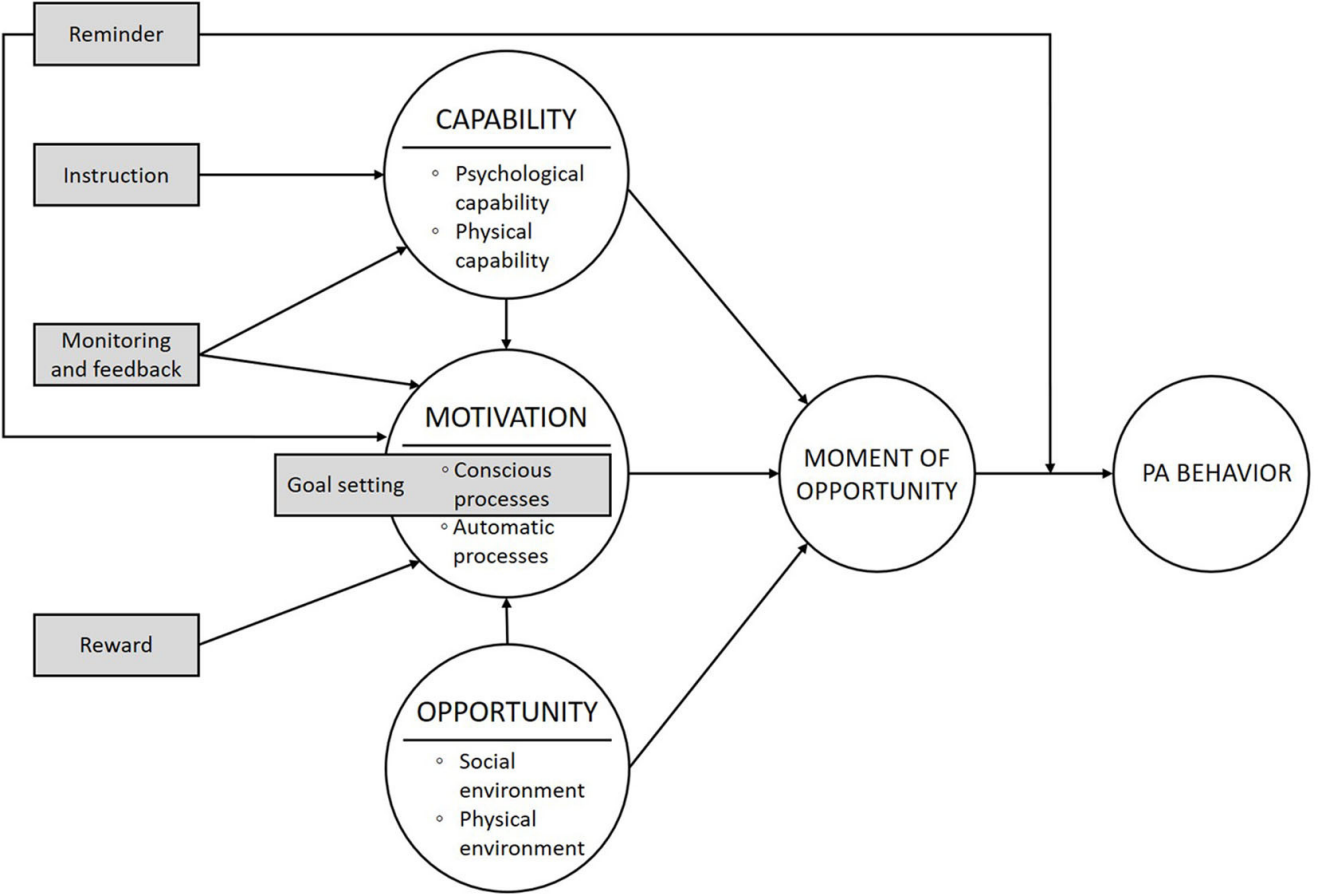
The hypothesis of how the persuasive strategies of the PAUL app influence the determinants of behavior.

Based on the findings of steps 1–5, we decided to include the strategy reminders, goal setting, monitoring and feedback, instructions, and rewards in the PAUL application. Based on several theories of behavior change [e.g., (27–30, 36, 38, 39)] and the BCW (25), we hypothesize that the persuasive strategies that are incorporated in the PAUL app influence the determinants of behavior in as indicated in Figure 3 (see step 4).

### Design the Functional Modules of PAUL App (Step 6)

In this step, we use the BIT model to describe “what” intervention modules the user receives (e.g., notifications) and how it relates to the persuasive strategies and “when” (e.g., under which condition) they have access to the module (24). In step 7, we explain “how” the modules look like (e.g., complexity and aesthetics). We defined these modules by drafting “user stories” with a team of stakeholders, behavioral scientists, computer scientists, and the app developer. The “user stories” served to translate the requirements into the app modules, that is, they explain the technical implementation and design characteristics of the selected persuasive strategies. An example of a user story is: “As a user, I want to restrict the amount of messages I receive during the week (max. 14 times a week), so I do not get disturbed too frequently and get annoyed by the intervention.” A brief overview of the operationalization of the strategies and the corresponding app modules is provided in Table 1.

**Table 1:** Overview of the persuasive strategies of the PAUL app, the app modules and a short description of the module.

**Persuasive strategy**	**App module**	**Short description of the module**
Reminders	Reminder module	• Timing of the reminders is adaptively personalized based on calendar availability, weather, the time of the day/day of the week, and previous PA behavior with the PAUL app. • Textual push notifications with a short and varying content.
Goal setting	Goal-setting module	• Each user sets a personal long-term PA goal for number of PA sessions/week and the duration of the PA sessions. • The system defines the (weekly and daily) short-term goals for the user based on the self-reported fitness level • The goals gradually increase in difficulty in order to reach the long-term goal by increasing PA time with ~10%. • Option to view and change the goal at all times.
Monitoring of behavior	Self-monitoring module of strength exercises	• After receiving a strength exercise prompt, the user has to log if she performed the exercise.
	Self-monitoring of outcome of behavior	• The user has the option to report notes about the training session, training intensity, and how satisfied she was with the training session.
	Automatic tracking module	• The app records and stores PA metrics during the app use (frequency, duration, speed, and distance). The user has to press “start” to initiate the behavior tracking. • The app also records and stores situational characteristics during each session and when sending a reminder (weather type, calendar availability, time, and date)
Feedback on behavior	Sustained feedback module	• The user can view simple on-screen feedback during PA and receives simple audio feedback.
	Cumulative feedback module	• The user can view a PA report immediately after the PA session with simple metrics such as distance and time (numerical). • The user can view a history consisting of all PA reports (including a map of the running route). • The user can view her progress toward weekly goal (home screen and goal-setting tag).
Rewards	Praise module	• Praise message after attaining the goal in on the landing page
Instructions	Instruction video module	• Instruction video to perform strength exercises (pushups and squats). • Triggered by a beacon during app usage. The user receives an audio tune and pop-up.

#### Reminders

The PAUL app sends a maximum of 14 reminder notifications each week. The content of the messages was drafted by 60 applied psychology students and were based on previous literature (45, 46, 61). To examine which type of message content individuals perceived as most motivating, a questionnaire was performed with 295 Dutch residents. The questionnaire consisted of 24 dual-choice items, four items in which the participants had to select the two or three best messages on a list of six or eight messages (respectively), and several items on socio-demographics and determinants of behavior. The results indicated that individuals prefer reminders that provides information regarding the progress toward their goal (in line with the findings of the focus groups) and messages that inform individuals on affective, immediate outcomes of performing PA (such as feeling happy). Furthermore, participants preferred messages in which the individual was addressed by name and that were positively framed (results not published).

Based on the outcomes of this study, a message library of 141 messages was built. The messages address the individual by name, are positively framed, focus on affective, immediate outcomes, and are tailored to the activity type (e.g., running, walking, and both). The messages can be classified into three different contents, namely, coping, feedback, and informative messages (see Table 2). Individuals have 50% chance to receive a coping message if they did not reach their goal the week before. In other situations, there is an equal chance for receiving a feedback or informative message.

**Table 2:** The three different content types of the PAUL reminders.

**Message type**	**Example**
Coping message	Will you go for the extra mile today? You were doing good [*name*]! Don't quit after a setback. Today is a new day. Will you go for a run today [*name*]? It can be disappointing when you do not reach your goal. But this happens to the best of us, so keep trying!
Feedback message	How would you feel about going on a walk [*name*]? Today, your goal is to walk [*n*] minutes! Are you going to get started to reach your goal today? Still [*n*] minutes to go this week, good luck!
Affective message	Hey [*name*]! Are you going for a walk today? You could be proud of yourself when you go! Will you make the effort today [*name*]? The most difficult part is to start running. Once you've started, it's fun!

The best moment for sending the reminders depends on the context and is person dependent. We developed a reinforcement learning module that optimizes this moment, using the response of the person while using the app. For initial training of the module and for finding the important context variables, we used a large dataset from a commercial partner in the project. This dataset contains around 440 K runs performed by over 10 K users with information about running performance, timing, and weather information (62). We combined this data set with geographical data sets to study the effect of environment variables. The results of this study showed that the most important variables were day of the week, time of day, and weather (63). Therefore, the optimization of the timing of the message was done depending on these contextual variables. Furthermore, the calendar availability was taken into account, since participants explained that they do not want to be disturbed when they are otherwise engaged (steps 2 and 5).

#### Goal Setting

A goal-setting module was designed in which users can set their long-term goal and get personalized short-term (weekly) goals assigned by the system that gradually increases until the participants reach their long-term goal. To set the goal, the user is guided through a short questionnaire (consisting of five to nine questions), in which the user can choose (1) her preferred activity type (i.e., running, walking, or both), (2) her long-term goal (i.e., PA session frequency and duration), and (3) her current fitness level (i.e., current PA frequency and their perceived fitness level). The user performs the questionnaire by herself. Based on this information, a rule-based system determines a personalized PA program that increases the weekly activity time with ~10% if the short-term goal is reached, until the long-term goal is reached. Individuals are invited to set a goal when they open the app for the first time and they can view and change it at all times.

An example of a long-term goal could be that the individual wants to walk three times a week for 30 min and run once a week for 30 min. Based on the current self-assessed fitness level in the questionnaire, the short-term goal that is provided by the algorithm could be to start walking three times a week for 15 min and run once a week for 12 min. If somebody is a more experienced runner or walker, the short-term goal will be set higher by the algorithm. If this goal is reached, the short-term goal for the next week will be ~10% more difficult.

#### Monitoring and Feedback

The app tracks the running and walking when the user presses “start” and it stops tracking after the participant presses “stop.” PA is measured with GPS and time recording. The user can actively track the amount of strength exercises by pressing “performed” (during the PA session), and she can report notes about the training session, training intensity, and how satisfied she is immediately after the PA session.

In the focus groups, participants explained that they prefer simple metrics. Hence, the PAUL app provides numerical feedback on the running or walking duration, distance crossed, average and/or current speed, number of strength exercises performed, progress toward weekly goal, and a visualization of their walking or running route. Audio feedback on the PA duration is given every 5 min during a PA session. Two types of feedback are provided, namely, (1) on-screen and audio feedback during PA [coined as “sustained feedback” (64)] and (2) a PA rapport [coined as “cumulative feedback” (64)]. The user receives the cumulative feedback immediately after her PA session, but she can also review all reports in the history view at all times and she can view her progress toward her goal on the landing page and goal-setting page.

#### Rewards

Since the participants in the focus group study in general favored simple rewards, simple praise messages were included in the app. When the weekly goal was reached, the user receives a positive message on the landing page of the app (“You have reached your weekly goal!”).

#### JIT Instructions

During running and walking, the user is prompted (audio, vibration, and pop-up) to perform strength exercises by a beacon at an exercise location. For each park (park Transwijk, Utrecht; Sloterplas, Amsterdam; and Oosterpark, Amsterdam), around 20 exercise locations were chosen by health professionals. In consultation with the municipality and neighborhood organizations, the beacons were only attached to lantern posts. Since participants indicated in step 3 that they do not like too many prompts too soon after each other, the prompt is sent once every 5 min, with a maximum of three per PA session. The user can accept, decline, or ignore the prompt.

The user receives a pop-up notification of the instruction if the phone is on lock screen. If the app is in foreground, the user receives a pop-up video. The video demonstrates the execution of a strength exercise in the same surroundings of the participant. Two types of exercises were included for each location, namely, squats and pushups. Thus, in total, more than 120 videos were uploaded in the app. The videos can be watched without internet connection because they are automatically uploaded when downloading the app. The videos are similar for all parks (except for a different surrounding); hence, all videos are (1) featuring the same instructor, (2) include a countdown, and (3) provide audio support (“bliepjes”). The user can view, review, pause, and stop whenever she wants.

### Technical Operationalization of the App Components (Step 7)

In this section, we describe how the modules described above are integrated into one system and the characteristics of the modules (e.g., aesthetics and complexity).

#### Hardware and Software Architecture

**Figure 4:**
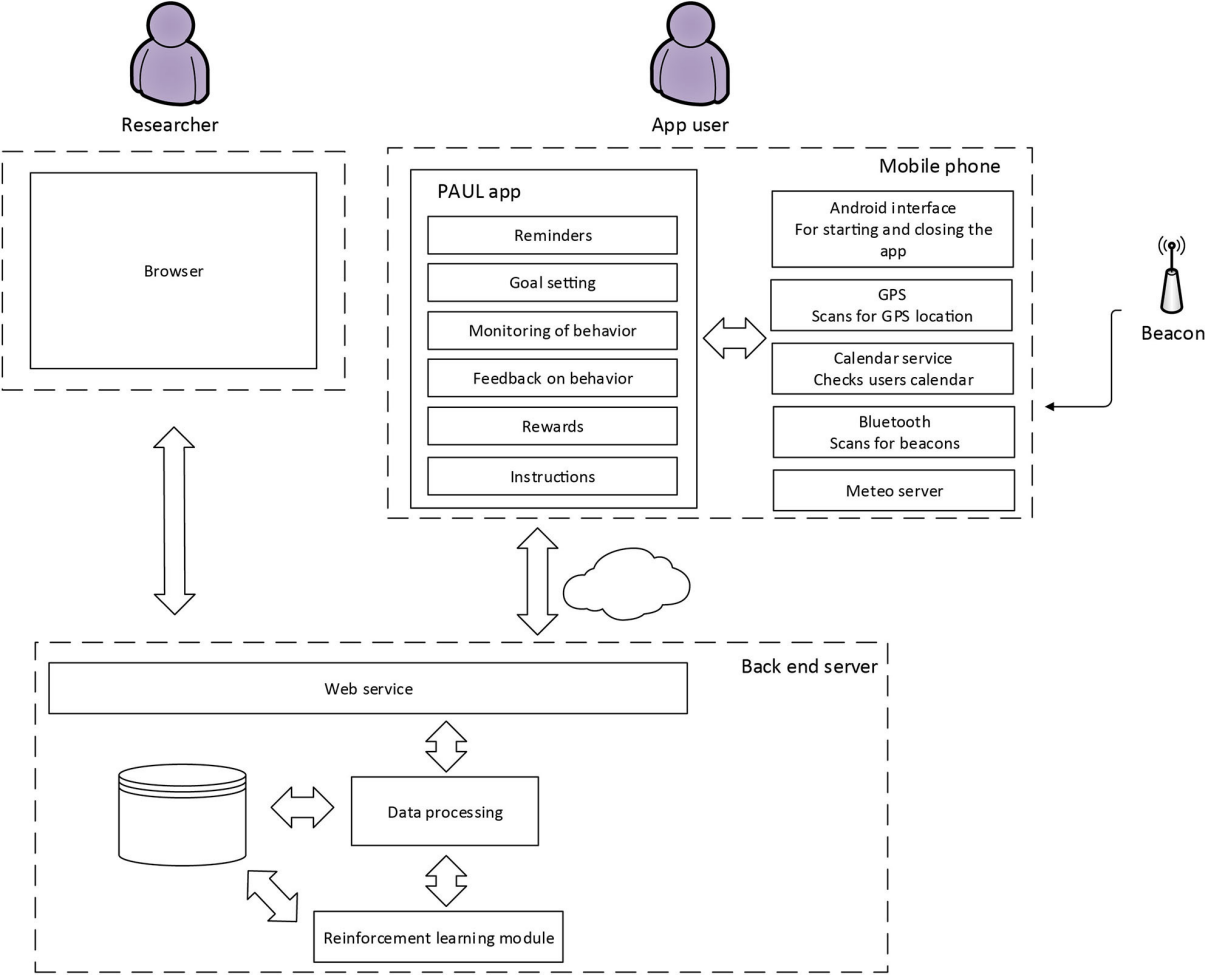
Software architecture of the PAUL app.

The modules have been implemented in a smartphone application. Since most Dutch residents use Android phones^3^, the app was built for Android. The app communicates with a remote server that collects data about app usage and makes it available for researchers within the project. The machine learning module for the adaptive reminders is also running on this server. Figure 4 gives an overview of the modules running on the different platforms.

**Figure 5:**
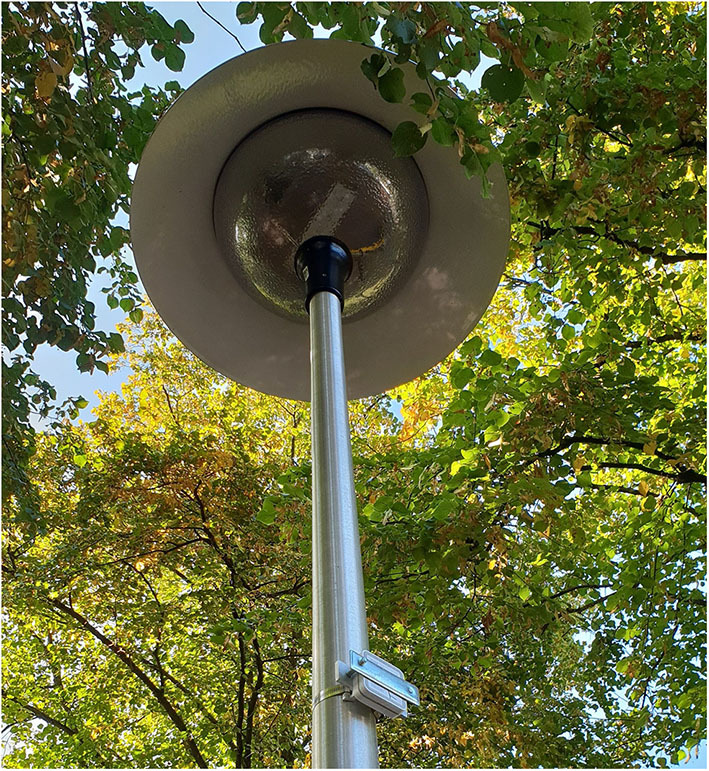
Beacon mounted in lamppost.

In order to provide the information about exercises that are to be carried out at specific locations, we used Bluetooth beacons (RedBeacon E4). It sends a Bluetooth signal every 100 ms that is received by the Bluetooth module on the smartphone. As mentioned in step 1, beacons were used since the municipality was interested in exploring how this technology can be used to increase PA behavior of individuals. The beacons are mounted unobtrusively, for example, in lampposts (see Figure 5), and can trigger a video in the app. As a failsafe, GPS was used.

#### User Flow

**Figure 6:**
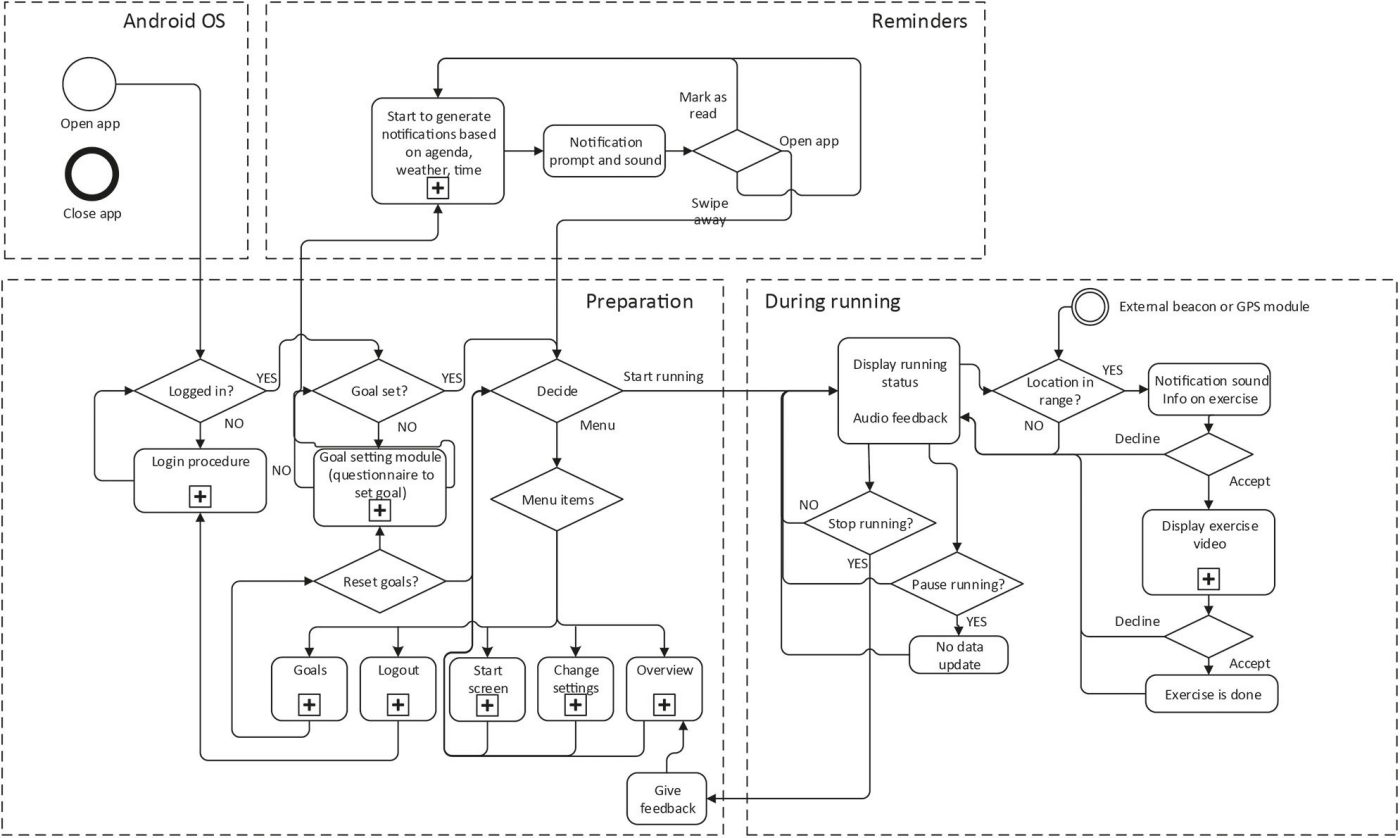
Navigation flow of the PAUL app.

On the smartphone, users can set goals, get feedback on their history and progress, as well as feedback on their momentary performance. The strength exercise instructions are also played on the smartphone. The reminders are displayed as a pop-up in the Android system. The user flow is shown in Figure 6.

#### User Interface

**Figure 7:**
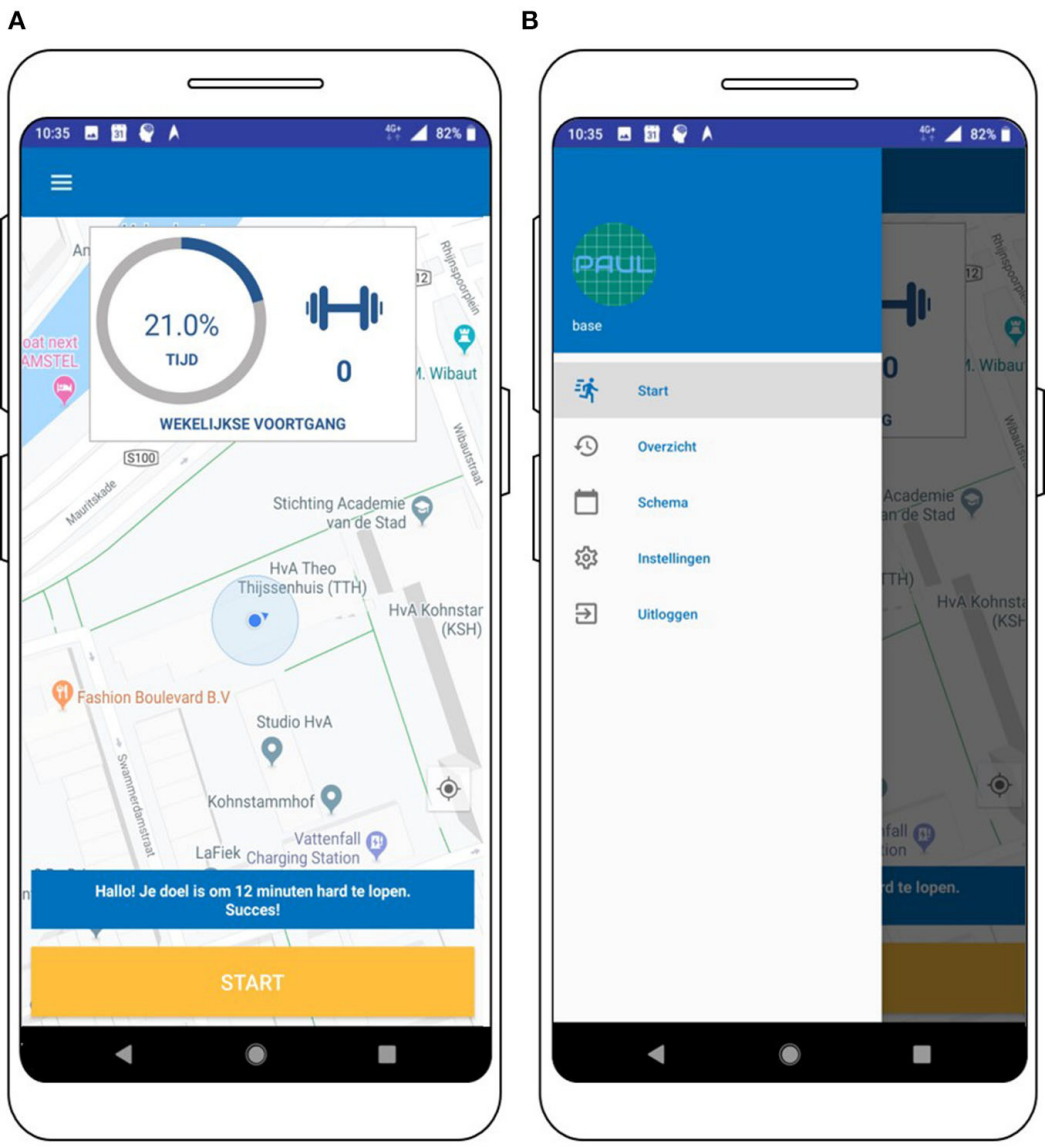
Screenshot of the landing page **(A)** and the menu of the PAUL app **(B)**.

**Figure 8:**
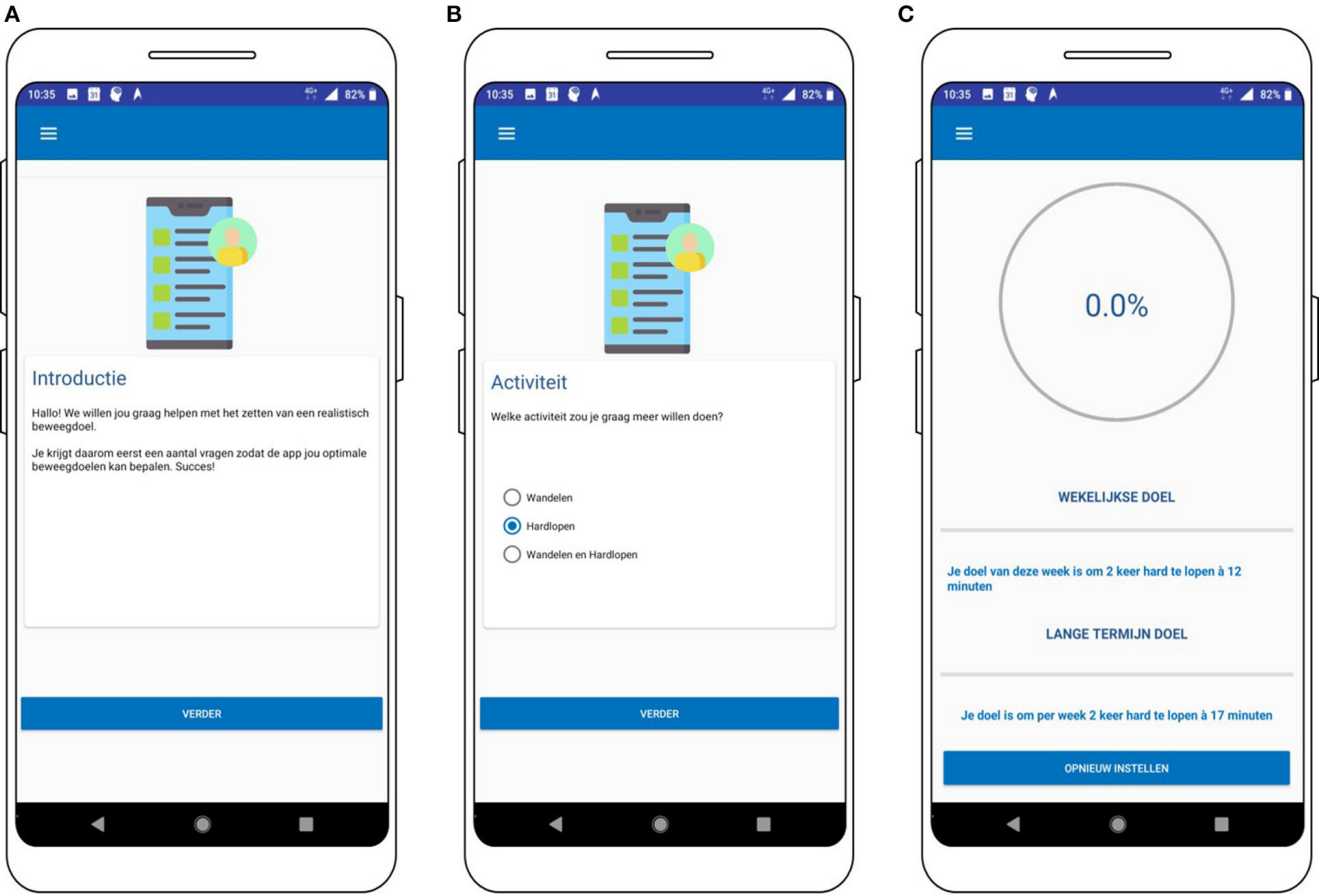
Screenshots of the goal setting module **(A,B)** and overview of the short- and long-term goal **(C)**.

**Figure 9:**
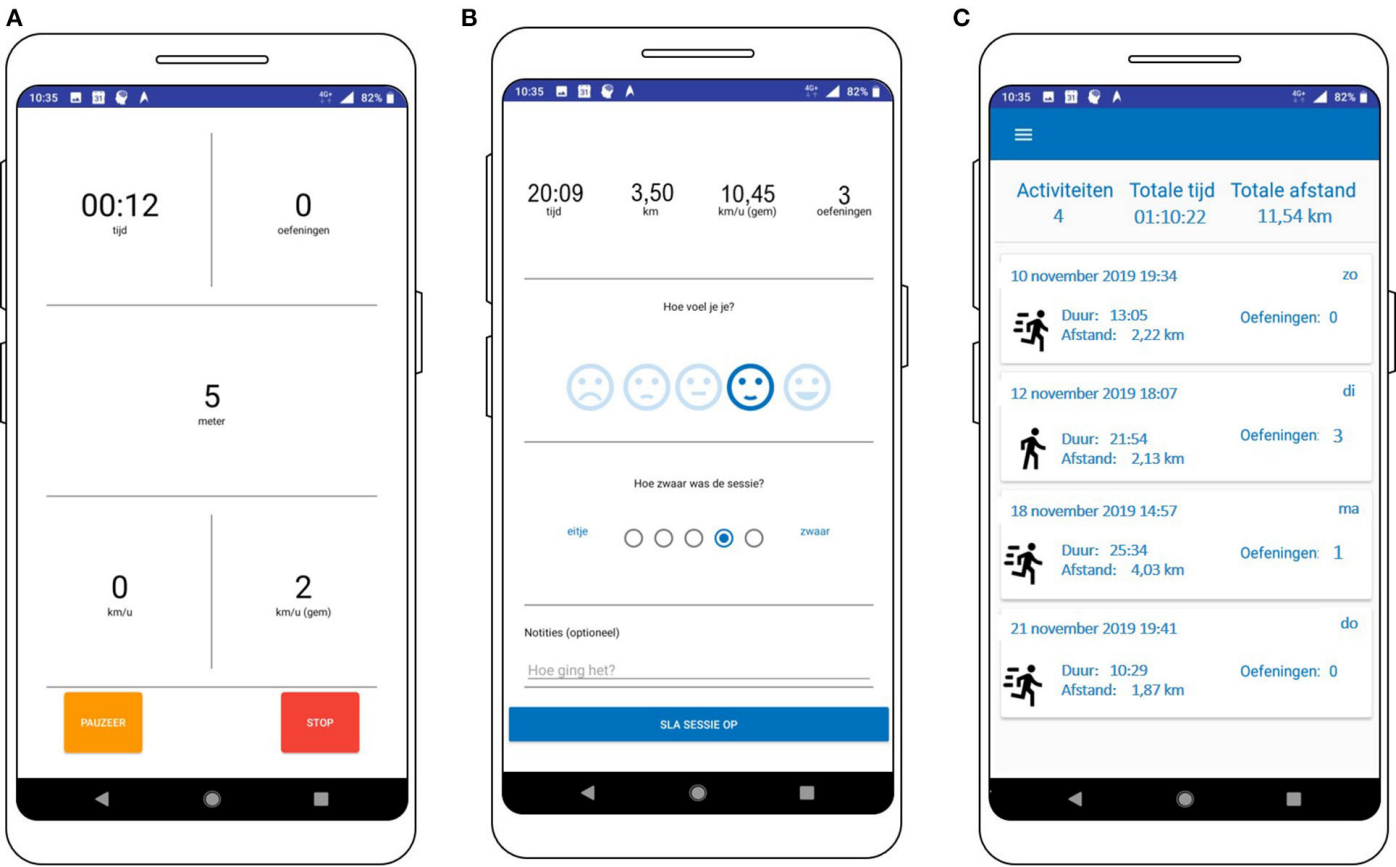
Screenshots of the feedback module during the PA session **(A)**, immediately after the PA session **(B)**, and the history view **(C)**.

**Figure 10:**
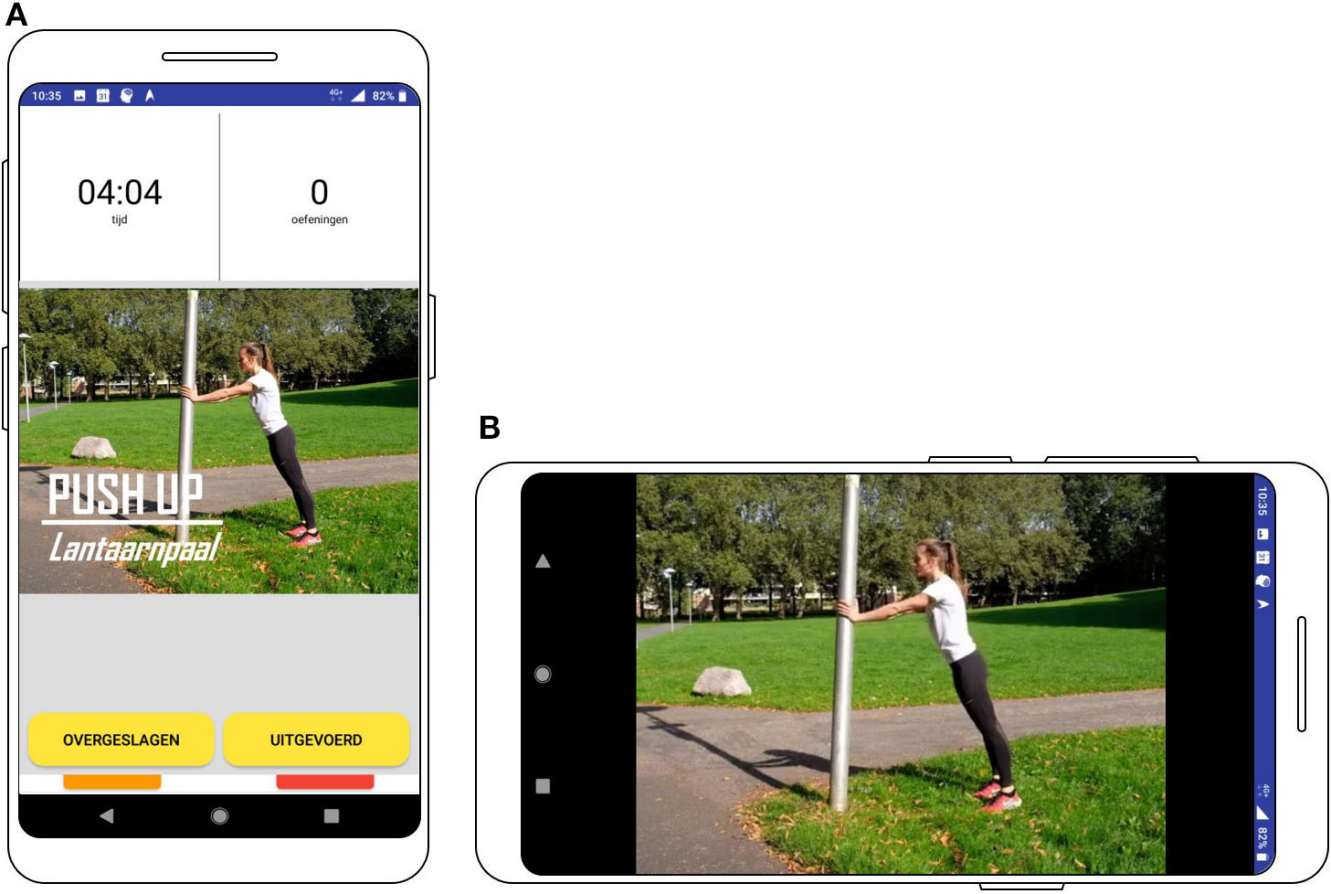
Screenshot of the prompt to perform the strength exercise **(A)** and the video **(B)**.

**Figure 11:**
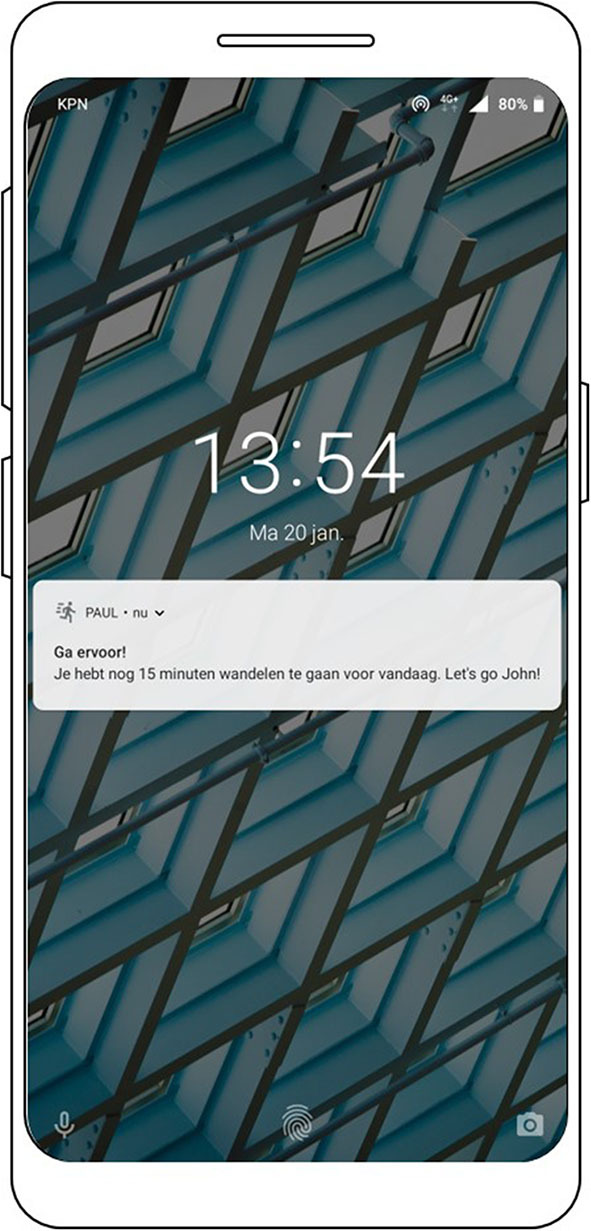
Screenshot of the reminder message.

Simplicity was a guiding principle in designing the user interface. Figures 7–**11** provide an impression on the resulting user interface. The usability of the app is increased by placing the “start” button at the landing page of the app (Figure 7A). By doing so, the user can start to track PA with just one click. Via the menu (Figure 7B), the user can navigate to the other modalities of the PAUL app.

For instance, the user can navigate to the “goal-setting module” by pressing “schema.” Here, the user can reset her goal by performing a simple questionnaire consisting of multiple-choice items (Figures 8A,B). Open questions were avoided since they might be too difficult and demanding for participants, while choosing between a few options is a relatively easy task. When the goal is set, the user can also view the goal in the goal-setting tab (Figure 8C).

During running or walking, the user can view sustained, numerical feedback whenever she wants (Figure 9A). The design is simple to ensure the most important feedback can be viewed with a quick glance. After finishing the PA session, the user immediately receives an overview rapport of her PA session (Figure 9B), where she can also easily monitor how she felt during the session and how heavy the session was by ticking the best suiting icon (Figure 9B). If the user wants to add more information about her PA session, she can enter this as well. The users can (re-)view their PA rapports as many times and whenever she wants, except during a PA session (Figure 9C).

The screenshot of a trigger to perform a strength exercise is shown in Figure 10. The user can choose to watch the video to get the information on where and how to perform the exercise. The video is displayed on full screen, but since some users might know how to perform the exercise or learns this over time, the user can also press “performed” to monitor that the strength exercise is performed, without having to watch the video.

The reminders are sent when the user has not performed a session or the amount of notifications that have been sent are below the daily reminder limit. Android has the ability to push messages to the screen of the phone. When a notification is sent, an icon will be visible in the phone's status bar and a notification sound will be played. The notification will also be visible on the lock screen of the phone when the screen has been locked (Figure 11). Android gives the user the option to selectively suppress notifications in general, per app, or per app channel depending on the version of Android running on the phone.

## Discussion

To gain a deeper understanding of why some mHealth interventions work, while others do not, mHealth researchers have been called upon to report a complete and detailed overview of the intervention components and its development process (20, 21). Therefore, this paper presents a detailed overview of the rationale, characteristics, and the development process of the PAUL app. The PAUL app aims to increase the perceived capability and motivation of individuals and to trigger the individuals “at the right time” to engage in recreational running, walking, and strength exercise behaviors, by levering on a self-regulation persuasive strategies. Novel app components are the optimization of the timing of the reminders via a self-learning algorithm and prompting real-time and location-specific strength exercises.

### Strengths and Limitations

One of the strengths of our methodology is the use of multiple frameworks in the app development, since they complement each other. The BCW was used to draft a hypothesis of how the selected persuasive strategies influence the (determinants of) PA behavior (25). With this conceptual model, it is possible to evaluate the working mechanisms of the PAUL app, i.e., if the application indeed changes the determinants of behavior and whether this leads to a change in behavior. Furthermore, as suggested by the IDEAS framework, end users were involved in the development to examine if the promising selection and operationalizations of the persuasive strategies that were identified in the literature are aligned with their needs and wishes. In addition to existing frameworks, we used data-mining techniques to guide the implementation of the personalized timing of the reminders. Data-mining technology has the advantage that already existing data can be used, reducing the participant burden. More importantly, the results of data-mining studies can provide a “warm start” for a self-learning system, which likely increases the effectiveness of these highly personalized applications (65).

There are also limitations to our study. First, although we did present our initial ideas of the PAUL app with screen shots to the users during the focus group study (step 5), we did not perform a study in which individuals could interact with the app, such as presenting a clickable design of the app to potential end users [cf. (66)]. Therefore, it is unknown if the app is easy to use and if users would have preferred a different interface. Since the usability and aesthetics of the app can influence the user engagement (67, 68) and app abandonment (69), we will explore this during a feasibility study (step 8).

A second limitation is that we only included individuals that lived close to the parks in Amsterdam. Although these individuals were recruited because they are the targeted users of the PAUL application, it limits the generalizability of our findings. For instance, it could be that people who live close to these parks (or other parks) might express a preference for bootcamp-like exercises, because they see more people performing these exercises in parks. Future studies could examine if similar technologies could be used by other target groups as well. In addition, there are a few limitations inherent to the use of smartphone technology for changing PA behavior, including internet connectivity, missing data, and the accuracy of the measurements [cf. (7)]. To reduce changes of malfunctioning, the instruction videos were already uploaded in the app. Thus, even if the participant had no internet access, she can receive and could watch the JIT exercise instructions. Furthermore, to increase the accuracy of the JIT exercise prompts, beacon technology was used. However, participants do have to turn on their Bluetooth to enable this function, which could deplete more battery. Lastly, for the application to track their PA behavior, participants do have to carry their phone with them during the run or walk, which could pose a barrier for the uptake of the intervention.

### Lessons Learned

During the development of the PAUL app, several lessons were learned of which future app developers and researchers could benefit. In regard of the app design, inactive individuals feel the need for smart and personalized intervention with variation that functions like a coach. Options for personalization should therefore not be limited to one app modality or persuasive strategy but rather across multiple app components. Furthermore, individuals also enjoy novel functionalities, such as the instruction video modality.

Regarding the methodology, we encountered three major challenges while developing the PAUL app. First, sometimes the theory and the user's wishes were not aligned. For instance, individuals explained that they liked the idea of the location-based bootcamp video's, while a recent literature review demonstrated that “information where and when to perform PA” or “instruction on how to perform the PA” were not related to positive intervention outcomes (8). A possible explanation is that the implementation of the intervention influences the effectiveness [cf. (43, 44)]. Thus, it could be that participants like instruction videos because they are new and easy to use, while other implementations, such as information sheets, are not effective. Another example is the goal difficulty; while individuals in step 5 explain they prefer an easy-to-achieve goal, the goal setting theory posits that difficult goals are more effective than easy goals to change behavior (29).

To address these contradictions, we discussed possible solutions within the research team. For the goal-setting module, we developed the module in which participants could receive both difficult and easy goals, depending on their own input. Thus, if they perceived their goal to be too difficult, they could make it easier. Regarding the instruction videos, we decided to keep the instruction videos based on the participants' enthusiastic responses during the first phase and because there are no studies that demonstrated that this operationalization of information is not effective (to the best of our knowledge). Notably, there is one research team that developed an mHealth intervention for running that includes strength training exercises which demonstrated positive results, but no effect study was performed (70, 71).

A second challenge was to cross the gap between the persuasive strategies to the operationalization of the strategies (i.e., the design characteristics and the technical implementation). Although the framework of (24) provides useful first design principles (such as tailoring the intervention), it does not provide a “do or don't” list regarding the operationalization. A promising method to make this translation is the use of more participatory methodologies, such as co-design or co-creation sessions [e.g., (72–74)], in which potential end users can (re)design the operationalizations of intervention components to their liking.

The third challenge relates specifically to developing JIT(A) interventions, namely, to account for potential delays of effects of the reminder on the behavior and the influence of prompts that are not sent by the application (either internal or external prompts). To illustrate, it could be that the reminder does not prompt the individual to engage in PA right away, but it does prompt the individual to make (or remind them to) the plan to go engage in PA when they get back home. Then, the actual cue to engage in PA would be “getting home.” It is therefore important to consider the time between sending and engaging with the message into account when evaluating the JITAI reminders. We aim to evaluate this possible delay in the ongoing feasibility study with the PAUL application by examining the user's perception and usage of the reminders with, for instance, interviews.

### Future Steps

Currently, the results of a 4-week mixed-methods feasibility study is being analyzed. The user experience with the app (such as user friendliness) was measured with a questionnaire, and semi-structured interviews were performed to examine the perception of the (operationalization) of the persuasive strategies of the participants. Since researchers are also called upon to explore if the mHealth interventions with similar theoretical groundings are feasible in a different cultural and environmental context (35), we are planning to perform a similar feasibility study in Brazil.

Based on the outcomes of the feasibility study, we will determine the next steps. If the app must be subjected to major changes, we will optimize the design and perform an additional small feasibility study. If the app is feasible, a large effect study will be performed to determine if the intervention increases the PA of individuals. In addition, we aim to investigate to what extent it is feasible to upscale the application. Currently, it's only functional at the three study locations, but other locations likely also support the performance of strength exercises. Therefore, we aim to examine the preferred locations (i.e., green spaces and sports areas) and amenities (i.e., benches, stairs, etc.) for performing strength exercises. This information can be used to determine more locations at which the exercise prompts can be sent.

## Data Availability Statement

The datasets for this article are not publicly available because the collected data (i.e., interviews) are privacy sensitive. No consent has been given to publicly share this data, but the data can be made available on request for verification purposes. Request to access the datasets should be directed to Karlijn Sporrel (k.sporrel@uu.nl).

## Ethics Statement

Written informed consent was obtained from the individual in the image for its publication.

## Author Contributions

KS, NN, and MS developed the conceptual model and performed the studies that involved the potential end users. RD built the PAUL app. SW performed the data-mining study and developed the self-learning module. MD, DE, BK, and VD supervised the project. KS and BK drafted the manuscript. All authors discussed the results and contributed to the final manuscript.

## Conflict of Interest

The authors declare that the research was conducted in the absence of any commercial or financial relationships that could be construed as a potential conflict of interest.

## Footnotes

^1^FBM uses different definitions of motivation then the COM-B model. In our work, we uphold the definition of the COM-B model. Additionally, the FBM does not use the concepts “opportunity” and “capability.” Rather, the FMB describes that the individuals should have the ability to perform the behavior, which is determined by both situations that lie outside of the individual and the characteristics of the individual herself.

^2^A beacon is a small device that sends out a Bluetooth signal, which can be recognized by an app. The app can then translate this signal to a meaningful message, such as an instruction video. Beacons have the benefit that they are more location specific than GPS.

^3^https://gs.statcounter.com/os-market-share/mobile/netherlands
